# Antimicrobial activity of *Origanum vulgare* L. essential oil and its effects on fungal gene expression

**DOI:** 10.1371/journal.pone.0329548

**Published:** 2025-08-01

**Authors:** Yan Li, Lu He, Zhiwei Xue, Yu Liang, Jiaying Jiang, Yiyan Wei, Huisi Li, Huimin He, Rongjun Li

**Affiliations:** 1 School of food and pharmaceutical engineering, Zhaoqing University, Zhaoqing, Guangdong Province, P.R. China; 2 Lushan Botanical Garden, Jiangxi Province and Chinese Academy of Science, Jiujiang, Jiangxi Province, P.R. China; 3 Shenzhen Qianhai Shekou Free Trade Zone Hospital, Shenzhen, Guangdong Province, P.R. China; Universidad Autonoma de Chihuahua, MEXICO

## Abstract

*Origanum vulgare L.* (oregano) essential oil showed strong antimicrobial potential, effectively inhibiting the growth of various microorganisms. In our study, oregano essential oil inhibited the growth of *Staphylococcus aureus* with a minimum inhibitory concentration (MIC) of 3.13 µL/mL and *Bacillus cereus* at 6.25 µL/mL. *Peronophythora litchii* growth was completely suppressed by oregano essential oil at a concentration of 12.5 µL/mL over a period of 7 days. GC-MS analysis identified carvacrol as the dominant component of the oregano essential oil. Transcriptomic analysis revealed that treatment of *P. litchii* with oregano essential oil resulted in 9,710 differentially expressed genes (DEGs) compared to the control. GO analysis indicated that these DEGs were primarily associated with glutathione metabolic processes, cytosolic ribosome function, and ubiquitin-like protein peptidase activity. Additionally, KEGG pathway analysis showed significant enrichment in proteasome, ribosome, and oxidative phosphorylation pathways. These findings demonstrate the potent antimicrobial efficacy of oregano essential oil against both bacterial pathogens and *P. litchii*, highlighting its promise as a natural antimicrobial agent.

## 1. Introduction

The fungus *Peronophythora litchii* (*P. litchii*) poses a significant threat to litchi quality, causing infections in leaves, flowers, and fruits during the pre-harvest stage, as well as litchi downy blight post-harvest, ultimately leading to significant economic losses [1,2]. Currently, chemical fungicides like metalaxyl and dimethomorph are commonly employed to manage *P. litchii* infections, however, their use raises concerns due to detrimental effects on the environment and food safety [3,4]. Therefore, it is crucial to develop natural sanitizers with antimicrobial properties that are non-toxic, enhance sensory quality, and extend the shelf life of litchi, aligning with both food industry standards and consumer preferences. Plant extracts are recognized as natural antimicrobial agents, offering safe and environment-friendly alternatives. For instance, apple polyphenols have demonstrated inhibitory effects against *P. litchii* [5]. Despite this, there remains a lack of effective and safe antimicrobial solutions to control *P. litchii* infections during postharvest storage.

*Origanum vulgare* L., commonly known as oregano, is an aromatic plant belonging to the Lamiaceae family. It is widely utilized in culinary applications for its distinctive flavor and aroma, as well as in traditional medicine for its digestive, anti-inflammatory, respiratory-soothing, and tonic properties [6,7]. The aerial parts of oregano are rich in a variety of bioactive compounds, including flavonoids, phenolic glycosides, terpenoids, and essential oils, which contribute to its diverse properties [8]. The essential oil, being the core chemical constituent of oregano, has been extensively studied in numerous research efforts. The drying methods applied to oregano significantly impact the concentration and composition of its essential oil, which in turn determines its suitability for specific applications [9]. Oregano essential oil has been demonstrated to possess a wide range of biological activities, making it a potential candidate for applications in skin repair and the treatment of inflammatory diseases [10]. Oregano essential oil also holds potential as a therapeutic agent for anticancer applications [11]. Additionally, oregano essential oil plays a significant role in the treatment of infectious diseases. It is employed to delay or inhibit the growth of pathogenic microorganisms due to its potent antimicrobial activity, primarily attributed to the presence of phenolic compounds like thymol and carvacrol, as well as hydrocarbons such as γ-terpinene and p-cymene [12,13]. For example, a low concentration of oregano essential oil demonstrated significant inhibitory effects against common foodborne pathogens such as *Staphylococcus aureus* and *Escherichia coli*. Additionally, oregano grown in different conditions exhibited variations in essential oil composition, which influenced its antibacterial activity against *Bacillus cereus* and *Bacillus subtilis* [14,15]. It also showed inhibitory effects on the growth of food-spoiling yeasts, positioning it as a potential alternative antimicrobial agent for food preservation [16].

Oregano essential oil is recognized as both toxicologically safe and effective in managing bacterial infections. Nevertheless, there is currently no available data on the effect of oregano essential oil on *P. litchii*. In the present study, we investigated the enhanced antimicrobial efficacy of oregano essential oil against *P. litchii*, with particular emphasis on transcriptomic-level variations induced by its antifungal action against *P. litchii*. This research offers a novel approach to extending the postharvest shelf life of litchi.

## 2. Materials and methods

### 2.1. Chemicals and pathogens

Oregano essential oil was purchased from Shanghai Yuanye Co., Ltd (Shanghai, China). Oregano essential oil was prepared by diluting with 0.5% aqueous Tween 80 to obtain a final concentration of 25 μL/mL. All reagents used in the study were analytical grade. The *Staphylococcus aureus* (*S. aureus* CMCC 26003) and *Bacillus cereus* (*B. cereus* CMCC 63302) strains were obtained from Guangdong Microbial Culture Collection Center (Guangzhou, China). The *P. litchii* strain was kindly provided by Dr. Bailin Li at the South China Botanical Garden.

### 2.2. *Evaluation of antimicrobial inhibitory effects of* oregano *essential oil*

The antibacterial activity of oregano essential oil was evaluated *in vitro* using the standard broth microdilution method. Bacterial strains were cultured in sterile Mueller Hinton broth and incubated at 37 °C for 24 h. The bacterial suspension was adjusted to a final inoculum density of 1–5 × 10^5^ cfu/mL based on optical density measurements at 600 nm. The methodology for evaluating antimicrobial activity followed the approach described by Li et al. [17]. To determine the minimum inhibitory concentration (MIC), the antibacterial activity of oregano essential oil was tested in sterile 96-well plates by a two-fold serial dilution, yielding final sample concentrations ranging from 0.78 to 25 μL/mL. Each well contained 50 μL of sample solution, 30 μL of nutrient broth, 10 μL of bacteria suspension, and 10 μL of resazurin solution (6 mg/mL), which was a redox indicator, providing a visual assessment of microbial viability. Control wells contained either 25 μM ampicillin (positive control) or 0.5% aqueous Tween 80 (blank). All assays were performed in triplicate. Additional 96-well plates were prepared with 50 μL of oregano essential oil and 50 μL of bacterial suspension, followed by incubation at 37 °C. Microbial growth inhibition was monitored every two hours by measuring the absorbance at 600 nm.

The antifungal activity of oregano essential oil against *P. litchii* was measured using the inhibition zone test, following a modified method reported by Xing et al. [18]. Oregano essential oil was added to potato dextrose agar (PDA) medium to achieve final concentrations of 0.78, 1.56, 3.13, 6.25, 12.5, and 25 μL/mL of the essential oil. In the control test, the essential oil was replaced with sterile water. After that, a mycelial plug (9 mm diameter) was cut from a purified culture of *P. litchii* grown on the PDA plate and placed at the center of new PDA medium containing the essential oil. Each treatment concentration was replicated three times. After an incubation period of 7 d, the inhibition zone around each mycelial plug was measured. Minimum fungicidal concentration (MFC) was defined as the lowest product concentrations which killed 99.9% of the inoculum [19]. For the determination of the antifungal activity, the size of the fungistatic circle was measured using a vernier caliper. The growth inhibition ratio was calculated according to the following formula: Antifungal activity (%) = (Dc − Dt)/ Dc × 100, where Dc (cm) was the mean colony diameter of the control sets and Dt (cm) was the mean colony diameter of the treatment sets.

### 2.3. *Determination of the components in* oregano *essential oil by GC-MS analysis*

For sample preparation, 100 μL of oregano essential oil was mixed with 900 μL pre-chilled *n*-hexane and vortexed for 30 s. The mixture was then subjected to ultrasonic treatment in an ice-water bath for 10 min. Following this, the samples were centrifuged at 12,000 rpm for 15 min at 4 °C. The resulting supernatant was transferred to a new vial for subsequent GC-MS analysis.

Analysis of components in oregano essential oil was performed using a SHIMADZU gas chromatograph coupled with a time-of-flight mass spectrometer employing electron impact ionization (70 eV). Separation of the main components in the sample extract (1 µL) was achieved using a DB-5MS capillary column (30 m × 250 μm × 0.25 μm). The sample was injected in splitless mode under the following operating conditions: the column temperature was initially held at 50 °C for 1 min, then increased to 310 °C at a rate of 8 °C/min, and maintained at 310 °C for 11.5 min. Helium was used as the carrier gas with a front inlet purge flow of 3 mL/min and a column flow rate of 1 mL/min. Mass spectrometry data was acquired in full-scan mode, covering a *m/z* range of 50–500, with a rate of 12.5 spectra per second after a solvent delay of 7.2 min. The experiment was performed in triplicate. The volatile compounds were identified by comparing their mass spectra with those in the NIST 21 library. The relative percentage of components in oregano essential oil was calculated based on the peak area.

### 2.4. Preparation of sporangia for transcriptome analysis

The spores and hyphae were rinsed with 10 mL of sterile saline containing 0.1% Tween 80. The mixture was then filtered through 4 consecutive plugs of sterile absorbent cotton wool to eliminate any hyphal fragments. The number of spores was counted by a hemocytometer and adjusted to a concentration of 1 × 10^5^ spores/mL to create a stock spore solution. For the preparation of spore solution, 100 μL of the stock spore solution was inoculated into 25 mL of potato dextrose broth (PDB) and incubated at 28 °C with shaking at 160 rpm for 96 h. To assess the inhibitory effects of oregano essential oil on sporangia germination, 100 μL of the spore solution was added to 10 mL of PDB medium containing 100 μL of oregano essential oil, resulting in a final concentration of 6.25 μL/mL for the treatment group. In the control group, sterile water was used instead of essential oil. Following incubation at 28 °C with shaking at 160 rpm for 48 h, the mycelial samples were washed three times with sterile water (15 mL each wash) and centrifuged (10,000 g, 4 °C, 10 min). The spores were collected and stored at −80 °C for RNA-Seq analysis.

### 2.5. Transcriptome analysis

Total RNA was extracted from the samples treated with oregano essential oil and sterile water using a Trizol reagent kit (Invitrogen, Carlsbad, CA, USA) following the manufacturer’s protocol. RNA quality was evaluated using a Qubit fluorescence quantifier and a Qsep400 high throughput biofragment analyzer. The RNA library for each sample was constructed and sequenced using an Illumina Novaseq by Metware Biotechnology Co., Ltd (Wuhan, China). For transcriptome analysis, clean reads were mapped, and low-quality reads that could interfere with assembly and analysis were filtered out to obtain unigenes. All unigene sequences compared with known sequences from the KEGG, NR, Swiss-Prot, GO, COG/KOG, and TrEMBL databases to obtain comprehensive annotation information.

### 2.6. Statistical analysis

The experiments were carried out in triplicate and the results were expressed as mean ± standard deviation. Differentially expressed genes (DEGs) were identified using thresholds of |log_2_Fold Change| ≥ 1 and false discovery rate (FDR) < 0.05. These DEGs were subsequently used for GO and KEGG enrichment analysis.

## 3. Results and discussion

### 3.1. Antimicrobial activity of oregano essential oil

**Fig 1:**
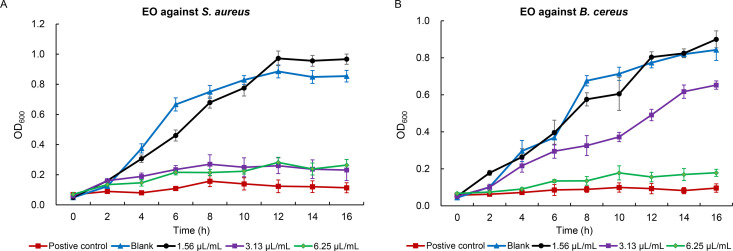
The inhibition curves of oregano essential oil (EO) on *S. aureus* (A) and *B. cereus* (B).

**Fig 2:**
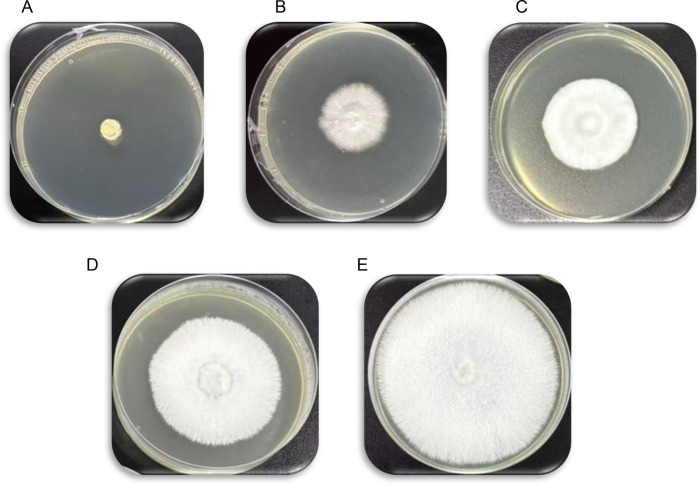
Antifungal activity of oregano essential oil against *P. litchii* was evaluated at different concentrations (A: 12.5 µL/mL, B: 6.25 µL/mL, C: 3.13 µL/mL, D: 1.56 µL/mL, and E: 0.78 µL/mL) following a 7-day incubation period.

The antimicrobial efficacy of oregano essential oil was assessed at concentrations ranging from 0.78 to 25 µL/mL. The results demonstrated that oregano essential oil exhibited significant antibacterial activity against *S. aureus*, with a minimum inhibitory concentration (MIC) of 3.13 µL/mL. Meanwhile, the MIC of oregano essential oil against *B. cereus* was 6.25 µL/mL (Fig 1). In addition to its antibacterial properties, oregano essential oil exhibited notable antifungal activity in a concentration-dependent manner. The MFC of oregano essential oil against *P. litchii* was determined to be 12.5 µL/mL, demonstrating complete (100%) fungicidal efficacy. Moreover, at a lower concentration of 6.25 µL/mL, oregano essential oil significantly reduced sporangia germination, achieving an inhibition of 65.6%. Further reductions in concentration of oregano essential oil resulted in progressively weaker antifungal activity: 53.1% inhibition at 3.13 µL/mL, 34.3% at 1.56 µL/mL, and no detectable inhibition (0%) at 0.78 µL/mL (Fig 2). These findings suggest that oregano essential oil, when applied at appropriate concentrations, can effectively inhibit the growth of *P. litchii in vitro*. Notably, complete inhibition of *P. litchii* was observed at a concentration of 12.5 µL/mL.

Previous studies have documented the antimicrobial efficacy of oregano essential oil against various fungal and bacterial strains, including *S. aureus*, as well as the fungi *Aspergillus flavus* and *Penicillium citrinum* [20]. The low MIC and MFC values further emphasize the potent antimicrobial properties of oregano essential oil. The overall results indicate that oregano essential oil possesses strong antimicrobial activity, making it a promising candidate for further exploration as a natural antimicrobial agent to mitigate the growth of *P. litchii*. Its efficacy suggests potential applications in significantly extending the shelf life of litchi.

### 3.2. Identification of chemical composition of oregano essential oil

**Fig 3:**
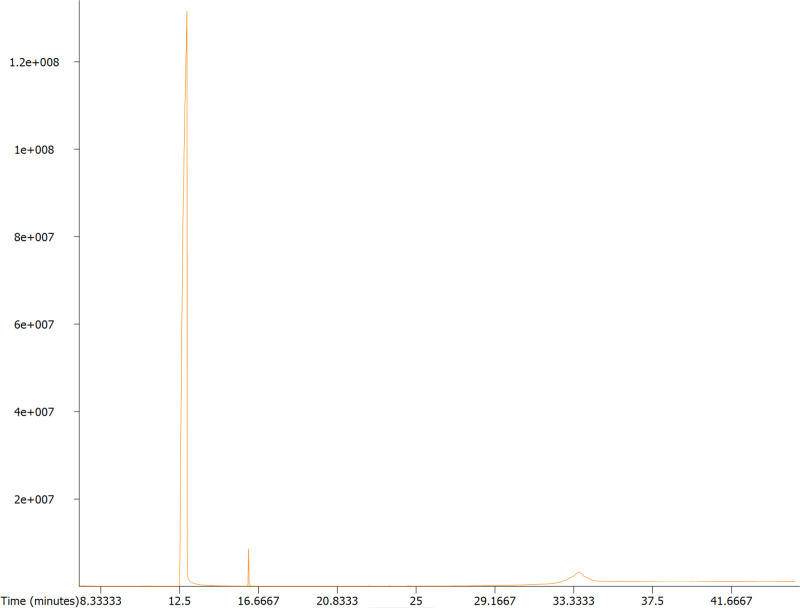
The total ion chromatogram (TIC) of oregano essential oil obtained from GC-MS analysis.

The chemical composition of oregano essential oil was analyzed by GC-MS, and the total ion chromatogram (TIC) is present in Fig 3. Carvacrol was identified as the predominant compound, eluting at 12.76 min and accounting for 99.89%. Additionally, signals corresponding to p-cymene and cuparene were observed at 7.33 and 16.07 min, respectively, both present in very low concentrations. Previous studies have also reported that the primary components of oregano essential oil are carvacrol, followed by p-cymene [21], which aligns with our findings. Oregano essential oil, which was rich in carvacrol, inhibited the cell viability of *S. aureus* ranging from 0.03 to 0.12 µL/mL [22]. Carvacrol also demonstrated significant antimicrobial activity, with MICs ranging from 0.05 to 0.15 µL/mL against *Listeria monocytogenes* strains [23], suggesting that it may be the key antimicrobial component in oregano essential oil.

### 3.3. Sequence assembly and annotation of transcriptome sequencing data

No germination of *P. litchii* sporangia was observed after exposure to 12.5 µL/mL of oregano essential oil, and the essential oil showed 65.6% growth inhibition of *P. litchii* at 6.25 µL/mL. Therefore, for transcriptome analysis, *P. litchii* treated with oregano essential oil at 6.25 µL/mL was used as the test sample, while *P. litchii* treated with sterile water served as the control. This assay aimed to identify the key molecular changes underlying the effects of oregano essential oil on *P. litchii*.

Transcriptome sequencing generated 57,659,851 and 56,837,854 raw reads for the treatment group and control group, respectively. After screening and filtering, 54,744,074 and 53,911,234 clean reads were obtained, accounting for 95% of the original reads (Table 1). Each library produced more than 8 G of clean reads, with a Q20 percentage exceeding 97%, a Q30 percentage above 93%, and the proportion of GC at 58%, indicating high-quality sequencing data.

**Table 1:** Statistical results of transcriptome sequencing from the treatment and control groups.

Sample	Treatment group	Control group
Raw Reads	57,659,851	54,744,074
Clean Reads	56,837,854	53,911,234
Clean Base (G)	8.53	8.09
Q20 (%)	97.76	97.86
Q30 (%)	93.28	93.47
GC Content (%)	57.75	57.84

The unigenes derived from RNA-seq were annotated against multiple databases, including KEGG, NR, Swiss-Prot, GO, TrEMBL, COG/KOG, and Pfam. A total of 39,215 unigenes were annotated across all databases (Table 2). Among these, 31,643 unigenes were successfully annotated in at least one database, accounting for 80.69% of all unigenes. The KEGG database had the fewest aligned unigenes, with 17,865, while the Pfam database had the largest number of annotated unigenes, totaling 29,743.

**Table 2:** The quantity and proportion of unigenes annotated in each database.

Database	Number of genes	Percentage (%)
KEGG	17,865	45.56
Nr	26,668	68.00
SwissProt	18,698	47.68
TrEMBL	26,193	66.79
KOG	21,637	55.18
GO	23,905	60.96
Pfam	29,743	75.85
Annotated in at least one database	31,643	80.69
Total Unigenes	39,215	100

### 3.4. Differentially expressed genes (DEGs) and enrichment analysis

**Fig 4:**
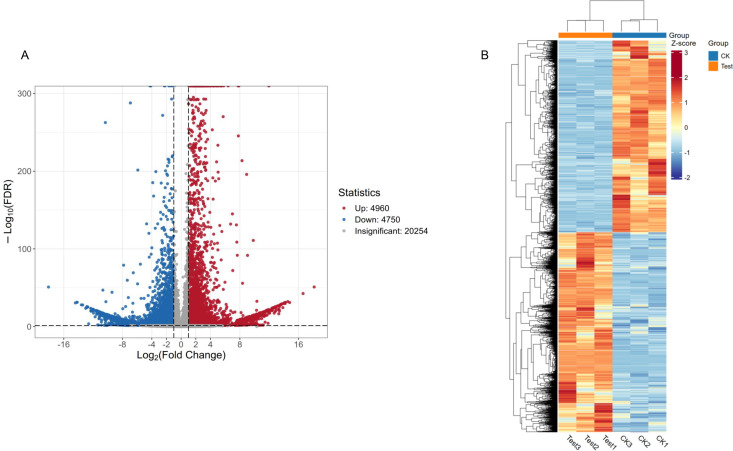
Differentially expressed genes (DEGs) were identified between the treatment and control groups. **(A)** Volcano plot showing the comparison of DEGs between the treatment group (Test) and the control group (CK). **(B)** Heatmap displaying the DEGs in the comparison of the Test and CK groups.

To examine the transcriptional differences between the treatment and control samples, we screened out DEGs via thresholds of FDR < 0.05 and |log_2_Fold Change| ≥ 1. Compared to the untreated group, the transcriptome data of the treated group suggested that 4,960 genes were upregulated and 4,750 genes were downregulated (Fig 4A). The heatmap of DEGs (Fig 4B) demonstrated a clear separation between the two groups, indicating that the gene expression changes were a result of the oregano essential oil treatment.

**Fig 5:**
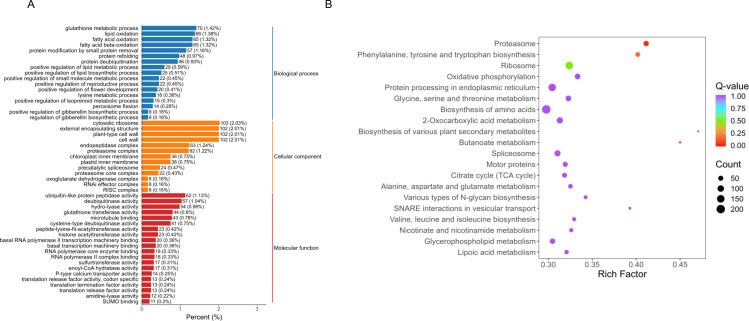
GO enrichment analysis (A) and KEGG enrichment analysis (B) of differentially expressed genes.

GO functional enrichment analysis was performed based on the DEGs (Fig 5A). Within the biological process category, DEGs were associated with ‘glutathione metabolic process’, ‘lipid oxidation’, ‘fatty acid oxidation’, and ‘fatty acid beta-oxidation’, highlighting distinct metabolic alterations between control and treatment samples. In the cellular component category, the most prominently enriched terms included ‘cytosolic ribosome’, ‘external encapsulating structure’, ‘plant-type cell wall’, and ‘cell wall’. For the molecular function category, DEGs were primarily linked to ‘ubiquitin-like protein peptidase activity’, ‘deubiquitinase activity’, ‘hydro-lyase activity’, and ‘glutathione transferase activity’. Together, these findings suggest that oregano essential oil treatment may disrupt cell wall integrity and associated cellular processes. Oregano essential oil has been shown to inhibit the activity of *Yersinia enterocolitica* by modulating processes related to cell membrane repair, energy supply and oxidative damage repair [24]. Similarly, its inhibitory effect on *Vibrio vulnificus* involves disrupting the cell membrane, decreasing intracellular ATP levels, and increasing reactive oxygen species and malondialdehyde concentrations [25]. These studies highlight the cellular component as a critical target mediating oregano essential oil’s antifungal effects against *P. litchii*. Additionally, DEGs encoding various enzymes were found to be significantly induced upon exposure to oregano essential oil. Further research has demonstrated that oregano essential oil exerts an inhibitory effect against *Salmonella Enteritidis* by interfering with enzymatic activities and disrupting DNA synthesis in bacterial strains [26].

We performed KEGG enrichment analysis and the most significantly enriched 20 pathway terms were shown in Fig 5B. The results revealed that the DEGs were primarily enriched in various pathways, such as ‘proteasome’, ‘ribosome’, ‘oxidative phosphorylation’, ‘protein processing in endoplasmic reticulum’, ‘biosynthesis of amino acids’, ‘spliceosome’, ‘motor proteins’, ‘citrate cycle’, ‘glycerophospholipid metabolism’, and ‘lipoic acid metabolism’. These enriched pathways are closely associated with key biological processes, including substance metabolism, signal transduction and genetic information processing. Research has demonstrated that oregano essential oil inhibits the growth of methicillin-resistant *Staphylococcus aureus* (MRSA) by damaging the cell membrane and disrupting enzymes involved in the tricarboxylic acid (TCA) cycle [27]. Our study also found that oregano essential oil had influence on the TCA cycle of *P. litchii*. Carbohydrates, glycerophospholipid, and various amino acids are essential components of cell structures, and their metabolic pathways were observed to be influenced by oregano essential oil. Previous studies have further indicated that the antibacterial activity of oregano essential oil against *S. aureus* involves disruption of ribosome function and interference with amino acid metabolism [28]. Collectively, these findings underscore the broad antimicrobial potential of oregano essential oil through its multifaceted mechanisms of action.

## 4. Conclusion

A low concentration of oregano essential oil exhibited remarkable inhibition efficacy on microorganisms, significantly inhibiting the growth of *P. litchii* at just 12.5 μL/mL. The primary inhibitory compound responsible for this effect was identified as carvacrol. In addition, the present study provides evidence for both potent antimicrobial effect of carvacrol-rich oregano essential oil and its potential role in gene expression modulation. The oregano essential oil demonstrated significant potential to disrupt microbial metabolism, particularly by impairing bacterial protein synthesis and amino acid metabolism. Given these findings, oregano essential oil may act as a natural preservative for extending the shelf life of litchi fruits. However, since this study did not evaluate its practical application in litchi preservation, future research should investigate the effects of oregano essential oil or carvacrol on litchi fruit storage.
